# Enhancement of light output power of GaN-based light-emitting diodes with photonic quasi-crystal patterned on p-GaN surface and n-side sidewall roughing

**DOI:** 10.1186/1556-276X-8-244

**Published:** 2013-05-17

**Authors:** Fang-I Lai, Jui-Fu Yang

**Affiliations:** 1 Department of Photonic Engineering, Yuan Ze University, 135 Yuan-Tung Rd, Jung-Li, Tao-Yuan country 32003, Taiwan; 2 Advanced Optoelectronic Technology Center, National Cheng Kung University, Tainan 701, Taiwan

**Keywords:** GaN, Light-emitting diodes (LEDs), Photonic quasi-crystal (PQC), Nano-imprint lithography (NIL)

## Abstract

In this paper, GaN-based light-emitting diodes (LEDs) with photonic quasi-crystal (PQC) structure on p-GaN surface and n-side roughing by nano-imprint lithography are fabricated and investigated. At an injection current of 20 mA, the LED with PQC structure on p-GaN surface and n-side roughing increased the light output power of the InGaN/GaN multiple quantum well LEDs by a factor of 1.42, and the wall-plug efficiency is 26% higher than the conventional GaN-based LED type. After 500-h life test (55°C/50 mA), it was found that the normalized output power of GaN-based LED with PQC structure on p-GaN surface and n-side roughing only decreased by 6%. These results offer promising potential to enhance the light output powers of commercial light-emitting devices using the technique of nano-imprint lithography.

## Background

Impressive recent developments of high-brightness light extraction of GaN-based nitride light-emitting diodes (LEDs) is dominated on both material techniques such as metal organic chemical vapor deposition (MOCVD) epitaxial growth and device fabrication processes. Thus, high-brightness LEDs have been used in various applications, including large- and small-sized flat panel displays backlight, traffic signal light, and illumination lighting by white light LEDs [1,2]. In order to get higher brightness of LEDs, extensive research has been conducted. One of the biggest problems in limited brightness of LEDs is the total internal reflection, which reduces the photon extraction efficiency of LEDs. Furthermore, the external quantum efficiency of GaN-based LEDs is low because the refractive index of the nitride epitaxial layer differs greatly from that of the air. The refractive indexes of GaN and air are 2.5 and 1.0, respectively. Thus, the critical angle at which light generated in the InGaN-GaN active region can escape is approximately [*θ*_*c*_ = sin ^− 1^(*n*_*air*_/*n*_*Gan*_)] ∼ 23*°*, which limits the external quantum efficiency of conventional GaN-based LEDs to only a few percent [3,4]. In order to avoid total internal reflection, various improving of the light extraction efficiency and brightness in the LEDs have been studied, including surface roughening texturing method [4-12], sidewall roughness [13,14], and insertion of two-dimensional (2D) photonic crystals (PhCs) [15-21]. All of these processes allow the photons generated within the LEDs to find the escape cone by multiple scattering from a rough surface, and a similar concept can also be applied to chip sidewalls. In other words, more photons should be able to escape from LEDs with surface patterned and textured chip sidewalls compared to LEDs with conventional flat chip. However, wet etching or nano-particle pattern with wet or dry etching used in most surface roughening techniques suffered the uniformity and reproduction problems.

In this paper, we report a feasibility of using nano-imprinting technique to fabricate patterned surface and sidewall of GaN-based LEDs for mass production. The nano-imprint technique is not only making well in controlling the nano-size coming truth but also highly reproducible. Hence, it is suitable for the mass production. Furthermore, only one pattern was used in this study to form structures in both top surface and sidewall region to combine the light enhancement effect of top and sidewall rough. The 12-fold photonic quasi-crystal (PQC) pattern was chosen as top and sidewall pattern owing to its capability to better enhance surface emission comparing with 2D PhC pattern approach [22]. Besides, according to the results of our previous work, the PQC pattern applied on the GaN LED could get more concentrated in the far field pattern of the GaN LED by comparing with the simply roughed surface and other extraction structures. As a result, the light output efficiency of LED with PQC structure on n-side roughing and p-GaN surface was significantly higher than that of a conventional LED. Additionally, the intensity-current (*L*-*I*) measurements demonstrate that the light output power of LED with PQC on p-GaN surface, LED with PQC on n-side roughing, and LED with PQC structure on p-GaN surface and n-side roughing was higher than that of a conventional LED at 20 mA with standard device processing.

## Methods

The GaN-based LED samples are grown by MOCVD with a rotating-disk reactor (Veeco, Plainview, NY, USA) on a c-axis sapphire (0001) substrate at the growth pressure of 200 mbar. The LED structure consists of a 50-nm-thick GaN nucleation layer grown at 500°C, a 2-μm un-doped GaN buffer, a 2-μm-thick Si-doped GaN buffer layer grown at 1,050°C, an unintentionally doped InGaN/GaN multiple quantum well (MQW) active region grown at 770°C, a 50-nm-thick Mg-doped p-AlGaN electron blocking layer grown at 1,050°C, and a 120-nm-thick Mg-doped p-GaN contact layer grown at 1,050°C. The MQW active region consists of five periods of 3 nm/7-nm-thick In_0.18_Ga_0.82_N/GaN quantum well layers and barrier layers.

**Figure 1:**
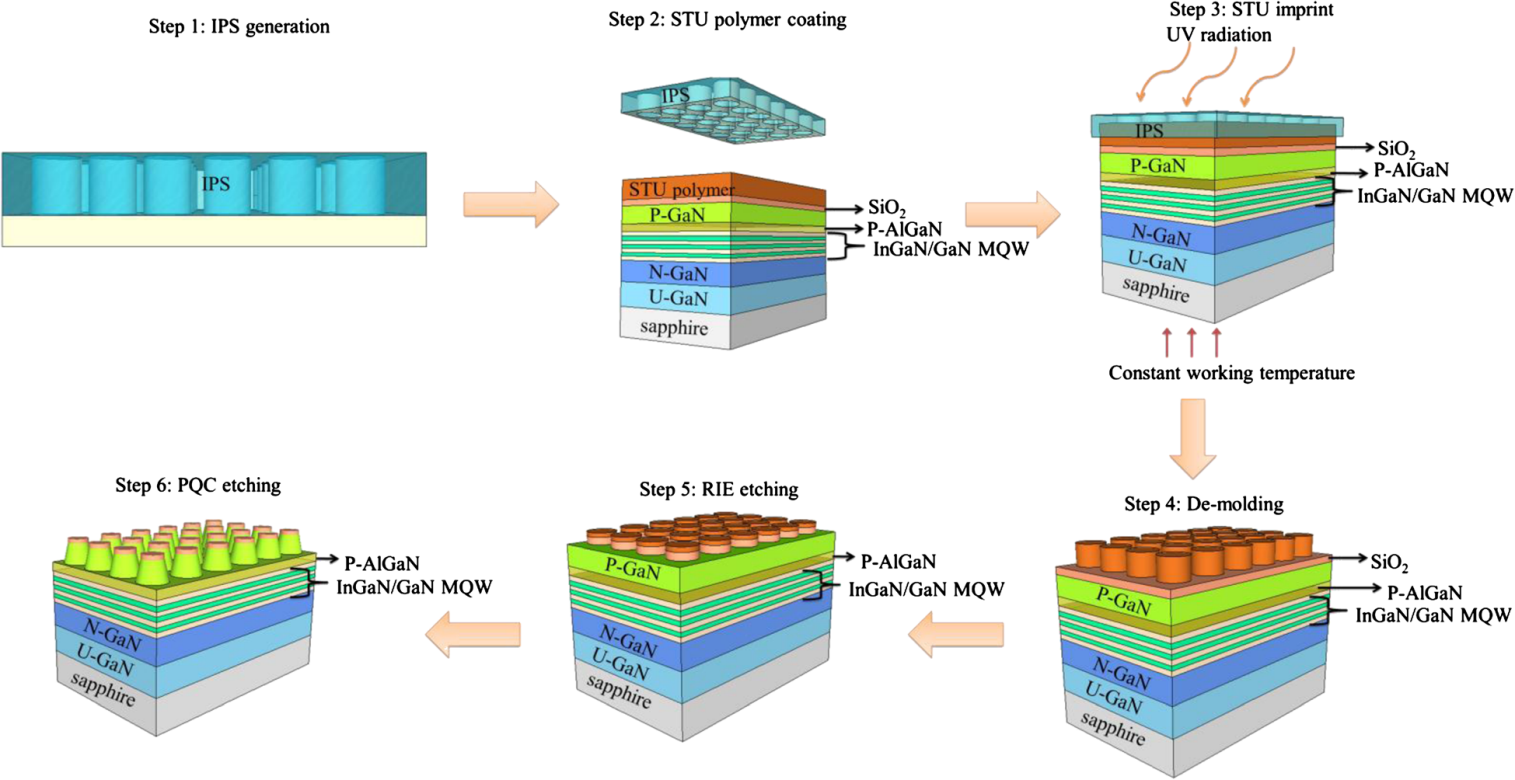
Schematic diagrams of GaN-based LEDs with PQC on p-GaN surface by nano-imprint lithography.

The detailed process flow of GaN-based LED with PQC structure on p-GaN surface by nano-imprint lithography is shown in Figure 1. The first nano-imprint step is generating a replication of an intermediate polymer stamp (IPS) from a Ni master stamp. Employing IPS stamps instead of hard stamps solves hurdles, such as (1) imprint at high pressures without damaging stamps or substrates, (2) imprint adaptively on non-flat surfaces or surfaces with particle contamination. Therefore, the soft material will not damage the master stamp or the substrate. It adapts to uneven surfaces such as epitaxial overgrown substrates or samples contaminated with particles. The pressure of 30 bar and a temperature of 160°C were applied to the nano-imprint lithography system for about 5 min. A 200-nm polymer layer was coated on the SiO_2_ (200 nm)/GaN LED sample surface at step 2, and these pre-polymers have thermoplastic properties, a very low glass transition temperature, and can be printed at temperatures ranging from room temperature up to 100°C. The pre-polymers have a sufficient number of reactive sites that can be activated for cross-linking by UV radiation, which takes place during a post-exposure bake that is executed at the same temperature as the other process steps.

Step 3 is in a simultaneous thermal and UV imprinting process, which is executed by the IPS imprinted on a pre-heated polymer layer. Applying a high pressure of 40 bar, the UV radiation time of 10 s and the constant working temperature of 65°C, the PQC pattern on the IPS can fully transfer to the polymer layer. In step 4, the LED samples and the IPS were then cooled down to the room temperature and release the IPS automatically. In step 5, the dry etching process of reactive ion etching (RIE) with CF_4_ plasma can remove the residual polymer layer and transfer the pattern onto the SiO_2_ film. The nano-imprint resin consists of a perfluorinated acrylate polymer and a photoinitiator. In step 6, we then used an inductively coupled plasma reactive ion etching (ICP-RIE) with BCl_3_/Ar plasma to transfer the pattern onto p-GaN surface.

**Figure 2:**
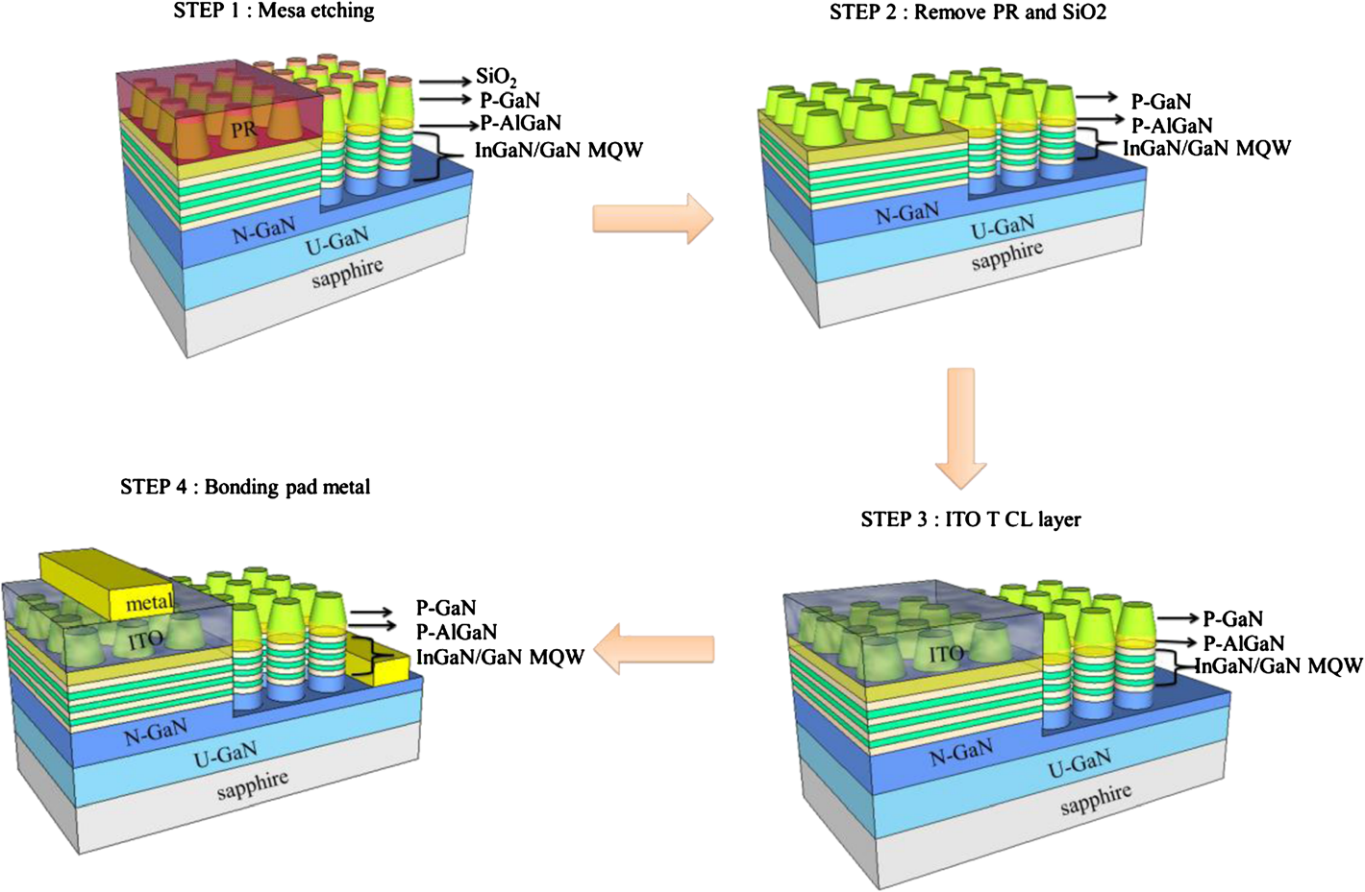
Schematic diagrams of GaN-based LEDs with PQC structure on p-GaN surface and n-side roughing process flowcharts.

A process flow schematic diagram of GaN-based LED with PQC structure on p-GaN surface and n-side roughing is shown in Figure 2. In step1, the LED samples with PQC on p-GaN surface and n-side roughing are fabricated using the following standard processes with a mesa area of 265 μm × 265 μm. A photoresist layer with thickness of 2 μm is coated onto the LED sample surface using spin coater, and the photolithography is used to define the mesa pattern. The mesa etching is then performed with Cl_2_/BCl_3_/Ar etching gas in an ICP-RIE system which transferred the mesa pattern onto n-GaN layer. In step 2, after the mesa etching, a buffer oxidation etchant is used to remove the residual SiO_2_ layer, and then, a 270-nm-thick indium tin oxide (ITO) layer is subsequently evaporated onto the LED sample surface in step 3. The ITO layer has a high electrical conductivity and a high transparency at 460 nm (>95%). In step 4, the metal contact of Cr/Pt/Au (30/50/1,400 nm) is subsequently deposited onto the exposed n- and p-type GaN layers to serve as the n- and p-type electrodes.

**Figure 3:**
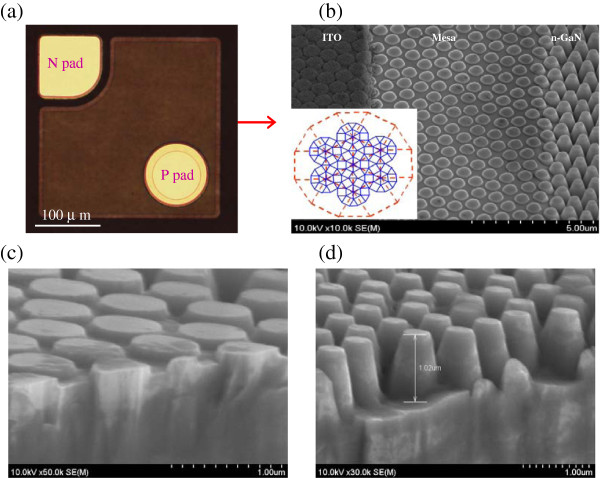
**Photos of LED surface.** (**a**) An optical micrograph of an LED die with PQC structure on p-GaN surface and n-side roughing, (**b**) the tilted plane view SEM image between TCL and n-side roughing region (left-side inset 12-fold photonic quasi-crystal model), (**c**) p-GaN surface, and (**d**) n-side roughing of cross section SEM images with photonic quasi-crystal structure.

Figure 3a is an optical micrograph of LED die with PQC structure on p-GaN surface and n-side roughing (LED chip area of 300 μm × 300 μm). The tilted plan view scanning electron microscopy (SEM) image between ITO transparent contact layer (TCL) and n-side roughing regions is shown in Figure 3b; the chip surface of GaN-based LED with PQC on p-GaN surface and on n-side roughing can be observed clearly, and further, the ITO film coverage on PQC nano-rod is uniform. The inset on the left side of Figure 3b shows the 12-fold PQC model based on square-triangular lattice.

The ‘photonic quasi-crystal’ is unusual with respect that on first sight, they appear random; however, on closer inspection, they were revealed to possess long range order but short range disorder [22,23]. The 12-fold PQC pattern was obtained from the PhCs with a dodecagonal symmetric quasi-crystal lattice than regular PhCs with triangular lattice and 8-fold PQC [22]. The recursive tiling of offspring dodecagons packed with random ensembles of squares and triangles in dilated parent cells forms the lattice. Additionally, the PQC rod dimension and pattern pitch were approximately 515 and 750 nm in this study according to [22] and roughly simulate calculation. Besides, dry etching depth of PQC structure was approximately 95 nm which was optimized through various depth etching, (the data is not shown here) since this etching depth could attain the best performance of light extraction efficiency of our LED structure from our etching test experiments. Figure 3c,d shows the p-GaN surface and the n-side roughing regions of cross section SEM images with PQC pattern, respectively. Further, the dry etching depth of the LED with PQC on n-side roughing was approximately 1.02 μm.

## Results and discussion

**Figure 4:**
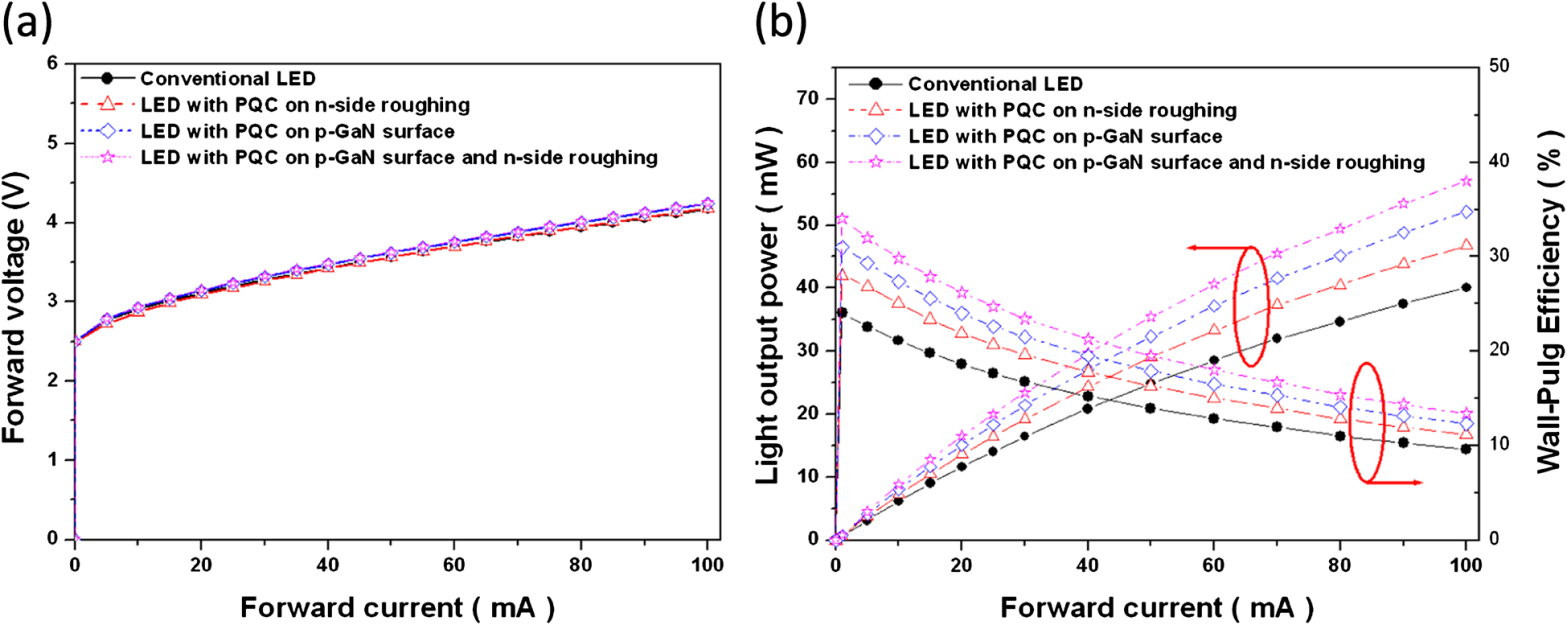
**Typical current–voltage (*I*-*V*) and light output power-current (*L*-*I*) characteristics.** (**a**) Current–voltage (*I-V*) characteristics of conventional LED, LED with PQC on p-GaN surface, LED with PQC on n-side roughing, and LED with PQC structure on p-GaN surface and n-side roughing, respectively. (**b**) Light output power-current (*L*-*I*) and wall-plug efficiency (WPE) characteristics of LED with/without PQC structure, respectively.

Figure 4a shows the typical current–voltage (*I*-*V*) characteristics. It is found that the measured forward voltages under injection current of 20 mA at room temperature for conventional LED, LED with PQC on p-GaN surface, LED with PQC on n-side roughing, and LED with PQC structure on p-GaN surface and n-side roughing were 3.11, 3.09, 3.14, and 3.15 V, respectively. In addition, the dynamic resistance of conventional LED, LED with PQC on p-GaN surface, LED with PQC on n-side roughing, and LED with PQC structure on p-GaN surface and n-side roughing are about 15.9, 16.7, 16.8, and 16.8 Ω, respectively. Therefore, in terms of dynamic resistance, there is no influence on this type of devices by incorporating PQC structure. The measured forward voltages at an injection current of 20 mA at room temperature obtain similar *I*-*V* curves for all types of LEDs on PQC etching depth in p-layer which was 95 nm. The coverage of ITO layer on p-GaN surface was uniform and no void defects on p-type contact, as the result in an ohmic contact in the contact area of the PQC structure on p-GaN surface, and the *I*-*V* curves of LEDs were almost similar while the etching depth of p-GaN surface was less than 95nm; however, the etching depth of p-layer was over 110 nm which indicated that there is heating and charging damages between ITO and p-GaN layer.

The light output is detected by calibrating an integrating sphere with Si photodiode on the package device. The intensity-current (*L*-*I*) characteristics of the LEDs with and without PQC structure are shown in Figure 4b. At an injection current of 20 mA and peak wavelength of 460 nm for TO (transistor outline) can package, the light output powers of conventional LED, LED with PQC on p-GaN surface, LED with PQC on n-side roughing, and LED with PQC structure on p-GaN surface and n-side roughing on TO can are given by 11.6, 13.5, 15.1, and 16.5 mW, respectively. Hence, the enhancement percentages of LED with PQC on p-GaN surface, LED with PQC on n-side roughing, and LED with PQC structure on p-GaN surface and n-side roughing were 16%, 30%, and 42%, respectively, compared to that of the conventional LED. The higher enhancement of LED with both PQC structures was scattering and guiding light from LED top surface and n-side roughing onto the LED top direction [14,21,24] to increase more light output power. In addition, the corresponding wall-plug efficiencies (WPE) of conventional LED, LED with PQC on p-GaN surface, LED with PQC on n-side roughing, and LED with PQC structure on p-GaN surface and n-side roughing were 19%, 22%, 24%, and 26%, respectively, which addresses a substantial improvement by the PQC structures on top surface and n-side roughing as well at a driving current of 20 mA. Comparing with the conventional LED, the WPEs of LED with PQC on p-GaN surface, LED with PQC on n-side roughing, and LED with PQC structure on p-GaN surface and n-side roughing were increased by 15.8%, 26.3%, and 36.8%, respectively, at an injection current of 20 mA, The enhancement of WPE of LED with PQC structure on p-GaN surface and n-side roughing is relatively high comparing with other researches [10,13,14,24,25], which is because the light emitted from LED scattered by top PQC pattern and guided onto the LED top direction by n-side roughing [22,23,26], therefore resulting in the enhancement of WPE.

**Figure 5:**
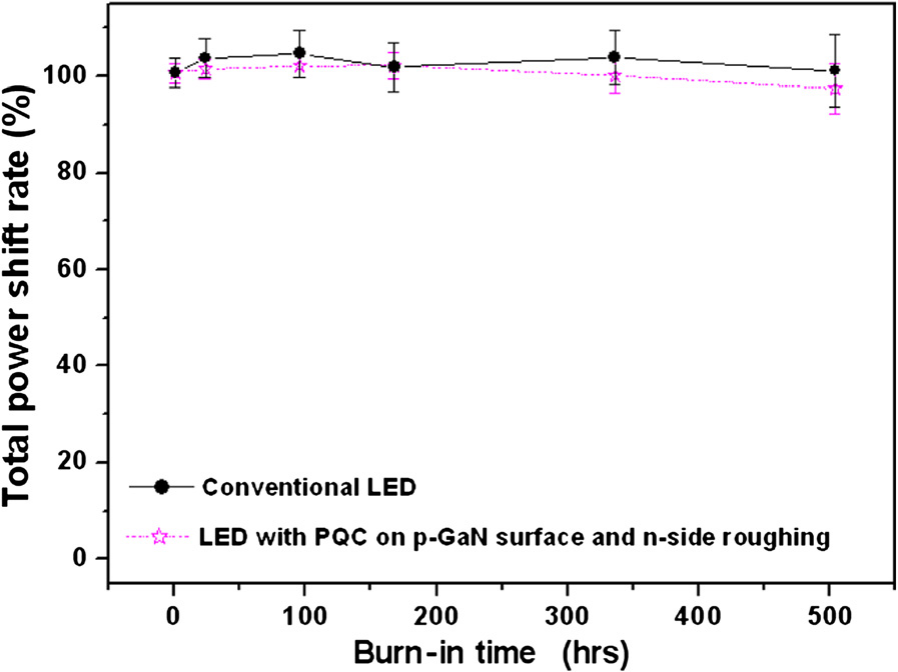
**The life test results of the conventional LEDs and LED with PQC structure.** The testing condition is under driving current of 50 mA and 55°C of ambient temperature.

During life test, 20 chips of conventional LEDs and LED with PQC structure on p-GaN surface and n-side roughing were encapsulated and driven by 50 mA injection current at 55°C of ambient temperature. As shown in Figure 5, after 500 h, it was found that the normalized output power of conventional LEDs and LED with PQC structure on p-GaN surface and n-side roughing only decreased by 6% and 7%, which indicates that the PQC structure is a reliable and promising method for device production. In general, the light output power of conventional type was decayed about 10% in aging test (55°C/50 mA), therefore indicating that the LED with PQC on p-GaN surface and n-side roughing did not damage the LED structure.

## Conclusions

The GaN-based LEDs with PQC structure on p-GaN surface and n-side roughing by nano-imprint lithography are fabricated and investigated. At a driving current of 20 mA on TO can package, the light output power of LED with PQC on p-GaN surface, LED with PQC on n-side roughing, and LED with PQC structure on p-GaN surface and n-side roughing were enhanced by a factor of 1.16, 1.30, and 1.42, respectively, and the wall-plug efficiency of the InGaN/GaN LED was increased by 26% with the PQC structure on p-GaN surface and n-side roughing. After 500-h life test (55°C/50 mA) condition, the normalized output power of LED with PQC structure on p-GaN surface and n-side roughing only decreased by 6%. This work offers promising potential to increase output powers of commercial light-emitting devices by using nano-imprint lithography.

## Competing interests

The authors declare that they have no competing interests.

## Authors' contributions

FIL carried out most of the experimental work including the material preparation and characterization and drafted the manuscript. JFY carried out the *L*-*I*-*V* measurements and the life test of PQC LEDs. Both authors read and approved the final manuscript.
